# The risks of perpetuating an evolutionary arms race in drug discovery

**DOI:** 10.1093/emph/eoz013

**Published:** 2019-05-21

**Authors:** Scott M Leighow, Justin R Pritchard

**Affiliations:** 1 Department of Biomedical Engineering, Pennsylvania State University; 2 The Huck Institute for the Life Sciences, University Park, PA, USA

## CHRONIC MYELOGENOUS LEUKEMIA

Chronic myelogenous leukemia is a leukemia of myeloid origin that is pathonognomonically associated with the production of the BCR–ABL fusion gene, a constitutively active tyrosine kinase, that can be treated with BCR–ABL kinase inhibitors [1].

Imatinib was the first inhibitor approved for use in BCR–ABL in 2001. However, as point mutations in BCR–ABL were identified, second- and then third- generation inhibitors were developed in an attempt to patch mutational vulnerabilities. The order of clinical use often follows the order of discovery [2].

Serially addressing the evolutionary failures of drugs locks us into an evolutionary arms race with cancer. The order of development is unlikely to be the optimal order for any malignancy.

## EVOLUTIONARY PERSPECTIVES

Cancer treatment leaves us with a unique evolutionary opportunity. Since every tumor arises *de novo*, we can ‘clean the evolutionary slate’ with each new diagnosis. What are the evolutionary consequences of applying drugs in the order they are developed?

**Figure 1.**
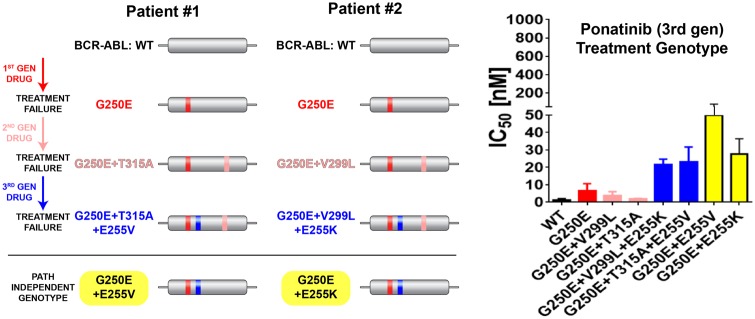
Left: A schematic of treatment progression and the genotypes tested for two patients. Right: Ponatinib IC_50_s measure 3rd gen inhibitor phenotypes. BaF3 cells with the indicated genotype were dosed (10 pts 3-fold dilution series in RPMI media, 3000 cells at *t* = 0, 72 h, *n* = 3, error bars are standard deviation). Note the complexity of genotype–phenotype relationships that were observed. This demonstrates that sequential inhibitor use selects for complex genotypes and creates evolutionary risks

Recently, we identified triple mutants in *cis* in the ABL kinase domain in two patients treated with a third generation inhibitor (ponatinib) who had failed two prior therapies [3]. The complexity of this treatment path led to a complex three mutant genotype where only the first and third acquired mutations were required for resistance to ponatinib (the third drug). A diversity of scheduling options creates multistep evolutionary changes encoding complex and redundant genotype–phenotype relationships (Fig. 1).

## FUTURE IMPLICATIONS

Imatinib and other kinase inhibitors have drawn reasonable criticism for their high prices. The recent approval of generic imatinib following patent expiration is economically exciting, but the order of approval of the generic versions of inhibitors will also follow the order of their discovery [4]. Thus, economics may incentivize patients and physicians to continue using drugs in the order they were developed.

A more evolutionarily principled treatment strategy is to utilize kinase inhibitors/combinations with fewer (and potentially no) vulnerabilities earlier. Such single agents and combinations have been developed, and are currently being tested for their ability to bring about better patient outcomes [5]. However, efficacy and safety may be challenging to achieve. Yet even if these trials prove successful, significant economic considerations may drive clinical practice towards repeating the experience of the last 18 years. Our challenge will be to consider the evolutionary impacts in light of the medical benefit and economic costs.
